# Analysis of Phenolic Compounds in Food by Coulometric Array Detector: A Review

**DOI:** 10.3390/s22197498

**Published:** 2022-10-02

**Authors:** Mutasem Razem, Yubin Ding, Ksenia Morozova, Fabrizio Mazzetto, Matteo Scampicchio

**Affiliations:** Faculty of Science and Technology, Free University of Bozen-Bolzano, Piazza Università 1, 39100 Bolzano, Italy

**Keywords:** phenolic compounds, coulometric array detector, electrochemical sensors, food sensors, antioxidants

## Abstract

Phenolic compounds are an important group of organic molecules with high radical scavenging, antimicrobial, anti-inflammatory, and antioxidant properties. The emerging interest in phenolic compounds in food products has led to the development of various analytical techniques for their detection and characterization. Among them, the coulometric array detector is a sensitive, selective, and precise method for the analysis of polyphenols. This review discusses the principle of this method and recent advances in its development, as well as trends in its application for the analysis of phenolic compounds in food products, such as fruits, cereals, beverages, herbs, and spices.

## 1. Introduction

Phenolic compounds are natural bioactive organic substances found in considerable amounts in fruits, vegetables, cereals, and other food products [1]. In recent decades, the scientific community has increased its interest in extracting and characterizing these natural compounds due to their antioxidant, anti-inflammatory, and radical scavenging properties [2]. Many studies have shown that the increased consumption of food products rich in phenolic compounds help decrease the probability of cancer and heart disease [3]. This was proven by the beneficial effects of the “Mediterranean diet”, which is based on high consumption of olive oil, cereals, wine, fruits, dried fruits, and spices. Consequently, the scientific community and controlling authorities need precise, dependable, sensitive, and cost-effective methods to analyze phenolic compounds. Several analytical methods are commonly used for the analysis of phenolic compounds.

### 1.1. Spectrophotometric Methods for the Analysis of Total Phenol Content

Total phenol content is generally measured using the Folin–Ciocalteau assay, which is an official standard method for some food products, such as wine [4]. The reaction is based on the reaction of phenolic compounds with the Folin–Ciocalteau reagent, a mixture of phosphotungstic acid (H_3_PW_12_O_40_) and phosphomolybdic acid (H_3_PMo_12_O_40_) in alkaline conditions, which results in the formation of chromogens, detected spectrophotometrically at 765 nm. The results are expressed in gallic acid equivalents according to the calibration curve [5]. Although the method is simple, inexpensive, and widely used in food science, it has several drawbacks. The measurement results can be altered by various other reductant compounds, such as organic acids, vitamin C, and amino acids, and the presence of Fe (III) [6]. Moreover, all spectrophotometric methods suffer from matrix effects due to the differing solubility of samples, redox potential, and pH differences. 

Another spectrophotometric assay for total phenol analysis is the Prussian Blue method based on the reaction of the phenolic compounds with hexacyanoferrate (III) (K_3_[Fe(CN)_6_] in the presence of ferric chloride (FeCl_3_) and chloric acid (HCl), leading to the formation of hexacyanoferrate (II) ion, [Fe(CN)_6_]^4−^, which further reacts with Fe^3+^, forming a blue metallic complex Fe_4_[Fe(CN)_6_]_3_. The reaction is monitored at 725 nm [7]. The Prussian Blue assay is less time-consuming and more selective compared to the Folin–Ciocalteau method but also suffers from the presence of interfering compounds. Moreover, the metal complex may precipitate in aqueous conditions; thus, the dilution of the samples should be adjusted before analysis.

Other spectrophotometric methods include the total flavonoids assay [8] and the Fast Blue BB assay with diazonium salt [9]. All these methods are used to estimate the total phenolic content, are not selective for single compounds, and suffer from matrix effects, interfering compounds, and difficulties due to pH and solubility differences. 

### 1.2. High-Performance Liquid Chromatography with UV-VIS and MS Detectors

Another widely used method for the analysis of phenolic compounds in food is liquid chromatography (LC) [10]. Many phenolic compounds can be analyzed using LC with UV–Vis or diode array detectors; however, they usually have low detection and quantification limits and require a long method optimization time for good chromatographic separation of peaks. 

Recently, the coupling of liquid chromatography with mass spectrometry has been widely employed in food analysis due to its high accuracy and selectivity. Tandem mass spectrometry is a versatile technique allowing identification, detection, and quantification of compounds present even in trace amounts [11,12]. Advances in technology have allowed for better analyte separations and higher resolution in mass spectrometers. High-resolution mass spectrometry alongside UV–Vis spectrophotometry allows for the detection, identification, and quantification of a wide range of food components, such as antioxidants, vitamins, fats, and proteins [13,14,15,16]. However, the amount of data provided by mass spectrometer detectors is often huge and needs complex data treatment for the selection of the compounds of interest. Moreover, UV–Vis detectors do not provide any information on the antioxidant activity of the analyzed phenolic compounds.

### 1.3. Electrochemical Methods

Electrochemical sensors are often applied to study antioxidants and phenolic compounds in foods. In recent years, so-called “electronic tongues” are gaining increasing popularity, especially in the field of food quality control [17,18,19]. An electronic tongue is defined as “a multisensor system, which consists of several low selective sensors and uses advanced mathematical procedures for signal processing based on the pattern recognition (PARC) and/or multivariate data analysis” [20]. The existing literature on electronic tongues is extensive and encompasses a combination of different sensors, such as electrochemical, potentiometric, conductimetric, optical, and piezoelectric transducers [17]. Electrochemical sensors generally monitor the current at a fixed or programmed modulated voltage (amperometry/pulse voltammetry) or the voltage at zero current (potentiometry), while voltammetry measures the relationship between the current and applied potential. 

Recently, a new electronic tongue based on coulometry has been developed. The coulometric array detector (CoulArray) consists of several coulometric electrodes deployed in series and independently poised at potentials between −1000 and +2000 mV. Coulometric detection is an absolute method, such that the peak area can provide a direct means of quantification through the direct relating of the area to the sample mass using Faraday’s law. 

The development of electrochemical biosensors has also allowed the detection of phenolic compounds from the environment. Oxidative enzymes, such as laccase, have been immobilized on the surface of a carbon glass electrode to detect catechol [21]. An electrical response is shown upon the oxidation of the targeted substance following reaction with the surface of the electrode, and this signal is proportional to the concentration of the phenolic compound. The electrochemical biosensor proved to have a low limit of detection (2.07 × 10^−6^ M) when compared with other electrochemical biosensors [22]. The use of carbon-based nanomaterial electrochemical sensors for the detection of phenolic compounds is simpler, and these sensors have a fast response and lower cost than common analytical methods [23]. The drawback of these biosensors is that not all have the capability to identify the specific phenolic compounds present in a substance.

This review describes the principle of detection and the applications of the coulometric array detector. The different types of phenolic compounds found in food are discussed. An overview of phenolic compounds detected in food and beverages using the CoulArray detector is presented in Table 1.

## 2. Principles of the Coulometric Array Detector

### 2.1. Description of the Coulometric Array Detector

Coulometry is an electrochemical method that measures the number of coulombs (total charge) reacted during the redox conversion of an analyte. Compared to other electrochemical methods (e.g., amperometric or voltametric methods), coulometry methods do not rely on mass transport current control to determine the concentration of redox compounds [24]. This means that the current intensity is measured, and the concentration of analytes can be derived from it based on Faraday’s law [25]. This is because the analyte’s conversion rate when passing through the electrodes is nearly 100%. Coulometry can be considered an absolute method that does not require chemical standards or calibration curves for quantification [26] 

Coulometry can be conducted in both constant current mode and constant potential mode. The constant current mode obtains the total charge directly from the period of reaction completion. This can be achieved with only one redox-active species or species with succinctly different redox potentials [24]. Constant potential coulometry measures the current created when the redox compounds pass through electrodes with set potentials. Then, to calculate the charge and achieve proper quantification, it is necessary to integrate the area under the curve. The constant potential mode is preferred due to the better control of the side redox reactions. It is characterized by high sensitivity and low detection limits and utilizes low background current methods, which have been applied for the detection of analytes with redox potentials [27].

### 2.2. Coulometric Array Detector Apparatus

**Figure 1:**
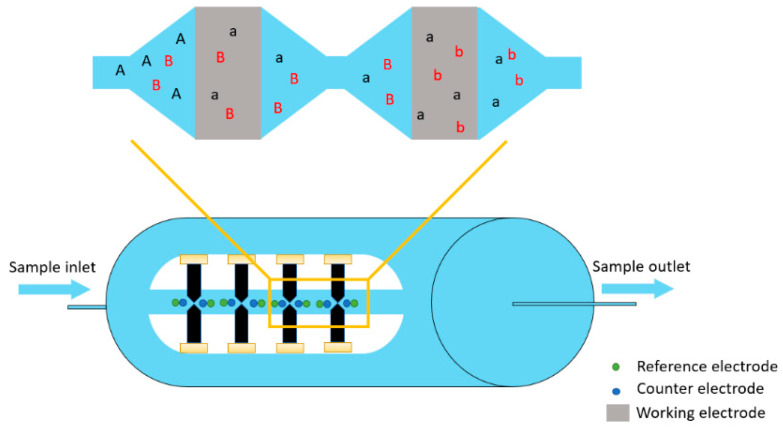
The inlet of the CoulArray detector. A and B represent compounds being eluted in the CoulArray detector, a and b are compounds who have lost electrons after being oxidized by passing through porous working electrodes set at different potentials.

The CoulArray is a commercial constant potential coulometry detector apparatus used for three-dimension determination of electroactive compounds. In a typical setup, four cell blocks are placed in series, each containing four porous graphite working electrodes, with platinum counter and palladium reference electrodes coupled to the apparatus (Figure 1) [28]. Up to sixteen coulometric cells are set at different potentials. In this way, for each sample, it is possible to collect an array of chromatograms that allow the identification of interesting compounds based on the retention time and oxidation characteristics.

The CoulArray can be applied in two modes, either connected with high-performance liquid chromatography (HPLC) or in flow injection mode. The flow injection mode allows fast measurements that can be applied for quality control purposes [29]. Morozova et al. designed a flow injection method to analyze the capsaicinoid content in habanero chili. The coulometric sensors poised at potentials between +400 mV and +450 mV showed the highest correlation with the capsaicinoid content (R^2^ = 0.95) based on 18 chili samples harvested from different types of soil [29]. The antioxidant capacity of lettuce can also be measured with a CoulArray detector, as described by Kongwong et al. [30]. From the 16 channels (potential ranging from +100 to +850 mV), the correlation between the signal detected between +400 mV and +750 mV was high (R^2^ > 0.97). The coulometric detector has also been applied to measure the contamination of the environment by pesticides in flow injection mode. The detection limit of chloride anions in the flow injection mode was 100 nM, with the 12 channels of the CoulArray poised at the same potential (−250 mV) [31]. The performance of the flow injection method based on the CoulArray detector was compared with that of a similar flow injection apparatus coupled with an amperometry detector. Ascorbic acid was used as the target analyte. Under optimal conditions (measured at +100 mV, 25 °C, 3:97 acetonitrile: acidified aqueous solution with 0.09% trifluoroacetic acid, flow rate of 0.13 mL· min^−1^), the detection limit of the CoulArray detector was 100 fmol (5 µL injection), which was considered better than the amperometry [32]. The flow injection method has also been applied to measure the oleuropein in herbs [33], and red wine [18] for the quantification of certain electroactive compounds or to connect their behavior with antioxidant capacity analysis.

**Figure 2:**
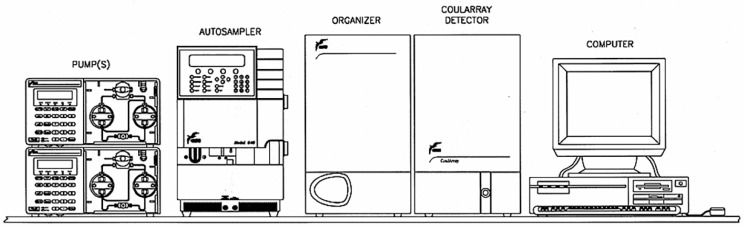
LC system with CoulArray detector (Thermo Scientific™ Dionex™ CoulArray™ coulometric array detector).

When the CoulArray detector is placed after an HPLC column, it can detect eluted bioactive compounds with enhanced selectivity, higher sensitivity, and lower detection limits (scheme shown in Figure 2). Moreover, this allows the possibility for the CoulArray detector to be used with gradient elution methods, unlike other electrochemical sensors that are limited to being used with isocratic elution.

The CoulArray has been applied across a variety of disciplines. Applications in the pharmaceutical area mainly focus on drug discovery and quality control. Hicks et al. have used the method to perform the absolute quantitation of pharmaceuticals with various functional groups of compounds. The system can also be applied to determine pipecuronium bromide and its impurities [34], macrolide antibiotics [35], and vitamins [36]. The CoulArray detector has been also studied with biological samples. For instance, it has been approved by the FDA for the determination of urine metanephrines and urine acid metabolites. Research by Luo et al. applied a CoulArray to analyze the oxidative DNA damage biomarker 8-hydroxydeoxyguanosine from urinary excretion [35]. Several other compounds, including bisphenol A [37], oxidative stress biomarkers (m- and o-tyrosine, 3-chlorotyrosine, and 3-nitrotyrosine) [38], and 5-(hydroxymethyl)-2-furfural [39], from biological fluids (urine, serum, or plasma) were also measured with the CoulArray. The CoulArray can also be applied to analyze food and beverages, including profiling characteristic bioactives, determining integrity, and identifying adulteration.

### 2.3. The Application of Hydrodynamic Voltammograms (HDVs) and Faraday’s Law

An HDV is a plot of the measured current versus the potential applied between the working and reference electrode. This is a sigmoid curve, which is often obtained from amperometry or cyclic voltammetry experiments [40]. However, since the CoulArray detector can detect the maximum peak current (and its charge) at a series of applied potentials, it can also generate an HDV plot. Furthermore, thanks to the coupling of this detector with HPLC systems, it is possible to obtain an HDV for each eluted compound. From the resulting current vs. potential plot, the half-wave potential can be derived. This is the potential value that is observed in connection with half of the total charge transferred by a compound. 

Currently, the CoulArray is mostly applied for the quantitative analysis of redox bioactives. The HDV plot’s additional information makes it feasible to distinguish the presence of co-eluted compounds, in addition to using the retention time to validate the presence of specific compounds [41]. Furthermore, a hydrodynamic voltammogram (HDV) and Faraday’s law can be applied for the analysis of the results from the CoulArray [40]. The HDV and the half-wave potential have been applied to verify compounds with standards and for the comparison of their antioxidant capacity [40]. 

In 2012, Webster et al. developed a method based on the flow injection CoulArray to select the suitable antioxidants needed for pharmaceutical applications. They determined that antioxidant additives can be detected if their half-wave potential is 100 mV lower than that of the pharmaceuticals. Based on this research, the CoulArray and HDVs can be applied to detect the electrochemical characteristic of both pharmaceuticals and antioxidant additives and, further, to choose appropriate drug formulations.

The redox compounds detected with the CoulArray can be quantified based on Faraday’s law (Equation (1)), where *Q* is the total charge accumulated from all the channels, *n* is the number of moles of electrons transferred during the redox event, *F* is the Faraday’s constant (96,485 coulombs per mole), and *N* is the injected analytes:

(1) Q=N×F×n

This equation is valid provided that the coulometric detector achieves nearly a 100% conversion rate when the sample is passing through the electrode. When such a conversion rate is reached, the concentration of the unknown analyte can be determined based on the injection volume. This approach was used to quantify pharmaceuticals by Hicks et al. and to quantify antioxidant compounds in officinal herbs by Ding et al. [25,42].

## 3. Phenolic Compounds in Food

### 3.1. What Are Phenols?

Phenolic compounds are secondary metabolites and derivatives of shikimate, phenylpropanoid, and pentose phosphate in plants [43]. Polyphenols provide protection against pathogens and contribute to the growth and reproduction of plants [44]. Moreover, phenolic compounds have a series of properties beneficial to human health, including antioxidant, antithrombotic, anti-inflammatory, and antimicrobial properties [45]. Phenolic compounds mainly have antioxidant effects due to their redox properties, which enables them to behave as hydrogen donors, singlet oxygen quenchers, and reducing agents. Phenols have also been reported to work as natural preservatives for foods, increasing their shelf-life [46]. Additionally, phenolic compounds have a wide range of commercial uses; for example, as natural food coloring agents or as paints, paper, and cosmetics ingredients. Due to these beneficial attributes, phenols have resulted in increased attraction among consumers.

**Figure 3:**
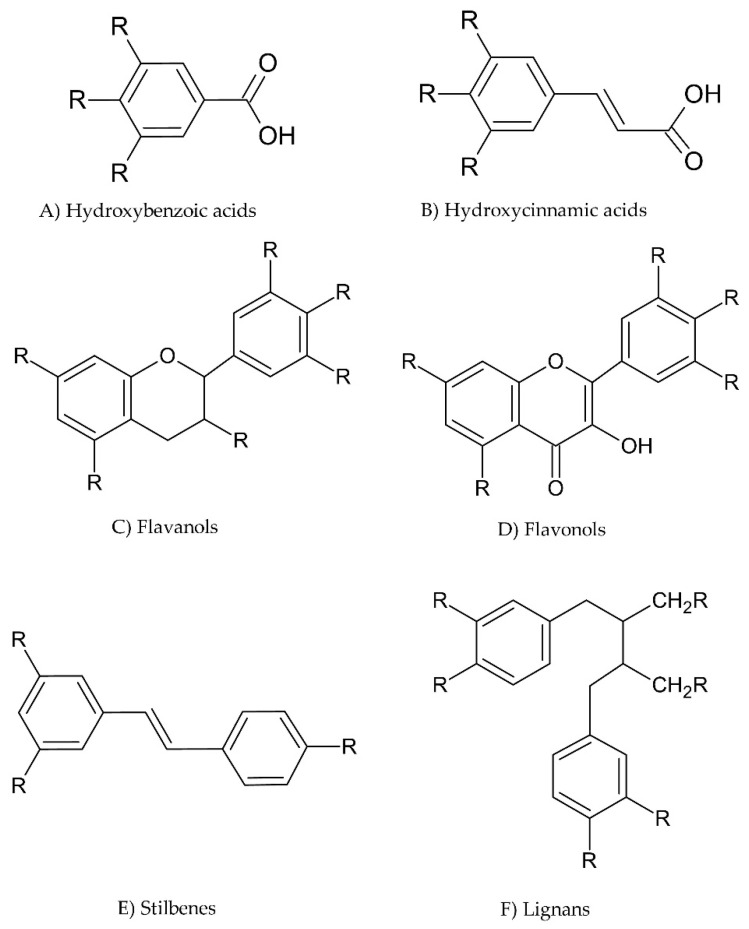
Chemical structures of common phenolic compounds.

There are over 8000 phenolic compounds that have been identified and the wide diversity has led to the sub-categorization of these compounds [47]. Phenolic compounds can be characterized by their origin, biological function, or chemical structure. These compounds contain an aromatic ring possessing one or more hydroxyl groups. Their classification depends on the number of phenol rings and the position of the hydroxyl groups. The most common classes of polyphenols are phenolic acids, flavonoids, lignans, tannins, and stilbenes [48]. Flavonoids are the most abundant group of phenolic compounds, and anthocyanins are derived from flavonoids, which are synthesized in plants through the phenylpropanoid pathway [49]. Some common examples of polyphenols are shown in Figure 3. Catechins are common flavanols that have all their functional groups as hydroxyl groups, as shown in Figure 3C. Phenolic acids are divided into two subgroups; hydroxybenzoic acids and hydroxycinnamic acids (Figure 3A,B). For example, if the functional groups in hydroxybenzoic acid are all hydroxyl groups, then we would have a gallic acid structure, while, in hydroxycinnamic acids, if only one functional group is present as a hydroxyl group, a coumaric acid is formed; however, if two functional groups are present as hydroxyl groups, then we would obtain caffeic acid. 

These phenolic compounds function as antioxidants by donating hydrogen atoms to reactive radicals and preventing the formation of free radicals [50].

Phenolic compounds exist either in free soluble forms or bound state forms [51]. The free or extractable polyphenols constitute only a small fraction of the total phenols present in foods or plants and bound polyphenols are more abundant than free phenols in foods [52]. Bound or insoluble forms of polyphenols often remain in the solvent used for extraction as their hydroxyl groups tend to link to macromolecules, such as proteins and polysaccharides; hence, giving them this name [53]. The main bound phenols present in plants are divided into two groups: condensed tannins and hydrolysable tannins. Condensed tannins are classified as flavanols and can be found in fruit seeds, plant stems, and fruit skins [54]. Hydrolysable tannins include polyphenolic acids or their derivatives, such as gallic or ellagic acid [55]. 

### 3.2. Food Sources of Phenolic Compounds

Phenolic compounds are naturally found in the environment and are present in plants, fruits, vegetables, herbs, oils, beverages, and elsewhere. Flavonoids are found in many vegetables, such as celery, cereals (barley), fruits (berries), and herbs [56]. Phenolic acids are found in apples, peaches, strawberries, and lemons. On the other hand, stilbenes are not as commonly found in the diet in high quantities, but they can be found in peanuts, red wine, and grapes [57]. Moreover, lignans are commonly found in cereals, such as wheat, barley, rye, and citrus fruits. By-products of agricultural and industrial residues are also a source of phenolic compounds [58]. Apple and pomegranate peels, for example, were used for the extraction of phenolic compounds for fortification into date bars for better nutritive value [59].

Beverages are an easy source of phenolics in the human diet. In the 21st century, many studies have been conducted related to the phenolic content present in wines, tea, and fruit juices, as some scientific evidence shows that drinking tea regularly can lower the chance of some types of cancer and cardiovascular diseases [60]. Wine is also an important source of antioxidants. Red wine, for instance, contains many flavonoid compounds that contribute to its color and have a total phenolic content value between 1724 to 1936 mg L^−1^, expressed as mg of gallic acid equivalents (GAE) [61]. Coffee is also known to be a rich source of antioxidant compounds, chlorogenic acid being the most dominant, with a total chlorogenic content of 5.26 mg g^−1^ to 17.1 mg g^−1^ found in a study by Fujioka and Shibamoto [60,62].

### 3.3. The Extraction of Phenolic Compounds in Food

Phenolic compounds are integrated into different food matrices and different extraction approaches are required to obtain them. The different chemical structures of phenolic compounds determine their solubility, along with the polarity of the extractant, if used. The main aspects of extraction rely on the type of extraction method, the polarity of the extract, the nature of the food matrix, and the analytical technique applied. Due to the diversity of phenolic compounds and their sources, a specific or standard extraction method cannot be applied. 

The most common or conventional method is solid–liquid extraction or leaching. The solid sample is in direct contact with a liquid matrix and a mass transport phenomenon occurs because of different aspects of the extract, such as the temperature, affinity, and diffusion coefficients [50]. The principle of this extraction method depends on the type of solvent used. Solvent extraction may produce a higher yield of phenolic compounds but, on the other hand, vast amounts of solvents may be needed, and the extraction time is long [63]. Solvents that are commonly used for the extraction of phenols from different foods are ethanol, methanol, water, ethyl acetate, and acetate [64]. Methanol and ethanol are the most common extractants used; however, there is limited choice in the type of solvent due to the complex matrices of food and the possible degradation of the targeted phenolic compounds [63]. 

Free phenols are soluble in organic solvents and can be extracted efficiently from their respective origins. On the other hand, bound phenols or insoluble phenols require a stronger solvent to break down the molecular structures and bonds between the bound phenols and the food matrix. This can be achieved through alkaline, acid, or enzymatic hydrolysis [65]. Alkali hydrolysis is the most common hydrolysis treatment used in cereals as acidic hydrolysis tends to degrade hydroxycinnamic and benzoic acids [66]. However, acidic hydrolysis may help release insoluble phenols, which are associated with the cell walls, polysaccharides, and some proteins. Sulphuric acid hydrolysis was used to determine and characterize polyphenols in different types of wheat bran and wheat flour [67].

The breakdown of the ester bonds between phenolic acids and the cell wall can be induced by a high pH with an alkaline solvent, which enhances the release of phenolic chemicals from polysaccharide structures. Horvat et al. used two molars of sodium hydroxide for the alkaline hydrolysis of barley, corn, and wheat [68]. Enzymatic hydrolysis is an environmentally friendly method that is easier to conduct compared to acid or alkali hydrolysis. Apple peels have been hydrolyzed using a range of different enzymes, including cellulases, ligninases, and pectinases [69].

## 4. The Application of the CoulArray for Analysis of Phenolic Compounds in Food

Phenolic compounds can be measured quantitatively using several reliable analytical methods, such as GC-MS and HPLC-MS. However, phenolic compounds exhibit antioxidant activity and they are considered electroactive compounds due to the hydroxyl groups present; alternatively, the use of electrochemical detectors (ECD) can help identify and quantify phenolic compounds from complex food matrices based on their redox potential and the number of electrons exchanged during the redox reaction. Electrochemical detection has high sensitivity, a broad linear measuring range, and low cost [70]. With the selective detection of antioxidants, the CoulArray detector can measure the different potentials of almost the entire analyte during the flow. The CoulArray detector’s response is unaffected by variations in the flow rate, temperature, and mobile phase composition. The use of multiple detectors allows a broad approach to characterizing the different phenolic compounds present in foods.

### 4.1. Fruits

The antioxidant activities of currant and gooseberry grown under an organic regime and in integrated pest management orchards were determined and compared by using the flow injection analysis method with a CoulArray detector [71]. The CoulArray consisted of four working porous graphite electrodes and reference hydrogen–palladium electrodes. The potentials of the electrodes were at +200, +400, +600, and +800 mV vs. the Pd pseudo-reference electrode, with the antioxidant capacity expressed as the electrical charge in C (Coulombs) per gram of fresh weight of fruit. The Triton black currant, grown in an organic orchard, had the highest antioxidant activity, with a value of 0.785 C g^−1^; the Jesan red currants had a value of 0.226 C g^−1^; and the Blanka white currant had a value of 0.356 C g^−1^.

Hajazimi et al. also determined different phenolic acids and flavonols in berries using the HPLC–CoulArray method [72]. Lingonberry, cloudberry, bilberry, and seabuckthorn berry are common berries consumed in the Nordic diet that contain a rich amount of dietary fiber; vitamins C, E, and K; polyphenols; and other bioactive compounds [73]. The flavonols quercetin, myricetin, kaempferol, and isorhamnetin and the gallic, vanillic, ferulic, *p*-coumaric, and caffeic phenolic acid aglycones of the berry species were determined. An ESA 5600A coulometric detector with eight porous graphite electrodes was used. The potentials that were applied were 0, +120, +240, +360, +480, +600, +720, and +840 mV. Sea-buckthorn berry contained the highest amount of the selected phenolic compounds with 270.5 mg/100 g DW, along with lingonberry with 219.7 mg/100 g DW. Cloudberry had the highest amount of hydroxybenzoic acid (66.8 mg/100 g), while bilberry demonstrated the richest amount of hydroxycinnamic acid (136.5 mg/100 g) and was especially abundant in caffeic and p-coumaric acids.

In a study carried out by Pyo et al., a reversed-phased HPLC coupled with a coulometric array detector was used to determine and characterize 13 different phenolic compounds from methanol extracts of Swiss chard (*Beta vulgaris* subspecies *cycla*) [74]. Standard solutions of nine phenolic acids and four flavonoids were prepared to compare with the Swiss chard samples and investigate their reproducibility and sensitivity. The sensitivity of the detection limit in this study was 1 ng mL^−1^. Moreover, the phenolic acids gave signals at low potentials (+70–+375 mV), excluding p-hydroxybenzoic acids, which responded at a potential of +825 mV vs. palladium reference electrodes. The total concentration of phenolics found in red Swiss chard was 157.8 mg/100 g FW, and in white Swiss chard it was 124.7 mg/100 g FW. Syringic acid was the most abundant phenolic compound found in both red and white leaf extracts. Table 1 indicates the different phenolic compounds found in different food items using a CoulArray detector.

The polyphenol profile of edible honeysuckle berries (*Lonicera edulis*) was also evaluated using a 12 channel CoulArray detector [75]. The honeysuckle is an uncommon fruit species mainly found in Russia that contains high contents of vitamin C and polyphenols [76]. The polyphenolic compounds that were discovered using the CoulArray detector were gallic acid, catalposide, rutin, resveratrol, quercitrin, chlorogenic acid, and quercetin, chlorogenic acid being the major antioxidant present (182 mg/kg FW), as expected by Jurikova et al. [77]. 

Strawberries (*Fragaria × ananassa*) are also known to have high quantities of phenolic compounds, mainly anthocyanins, as these are responsible for the red color of the fruit [78]. An eight-channel CoulArray detector with potentials set from +100 to +800 mV in increments of +100 mV was used to characterize the polyphenolic profile of strawberries [79]. The responses of the compound concentrations were plotted versus the oxidation potential in hydrodynamic voltammograms (HDVs) to characterize the polyphenols present. The selected phenolic compounds found in strawberry fruits included catechin, *p*-coumaroylhexose, and hydroxybenzoylhexose.

### 4.2. Herbs

Since ancient times, herbs have been used for culinary and medicinal purposes as they can prevent chronic diseases [80]. This is mainly due to the presence of phytochemicals with high antioxidant activity. Alkaloids, polyphenols, and carotenoids have been found in numerous officinal plants [81]. The antioxidants present in these plants can be evaluated using specific assays, such as 2,2-diphenyl-1-picrylhydrazyl (DPPH), ferric-reducing antioxidant power (FRAP), and oxygen radical absorbance capacity (ORAC) assays [82]. For better characterization and selectivity of the antioxidant species present, the coupling of HPLC with coulometric array detectors (CADs) has been applied. 

Ding et al. screened several antioxidant compounds in 19 officinal plants using HPLC-DAD-CAD-MS, with the CoulArray detector consisting of 16 porous graphite cells increasing from −50 to +700 mV with +50 mV increments [42]. The use of 16 electrodes in series resulted in an increase in the dynamic range of the CoulArray detector. *Moringa oleifera* was used to compare the antioxidant compounds with the other officinal plants due to it being a rich source of phenolic compounds. The most dominant antioxidant species present in the *Moring oleifera* extract was hyperoside, followed by neochlorogenic acid. With the use of Faraday’s law, the concentration of each antioxidant compound was determined. The total charge (Q_tot_) of each plant extract was obtained by summing up all the 16 channels of the CoulArray detector, as the total (Q_tot_) indicates the total antioxidant capacity of each plant extract. The *Melissa officinalis* and *Fraxinus excelsior* herbs had the highest Q_tot_ at 1900 and 2646 µC, respectively. The CoulArray detector was also used to determine phenolic compounds in several aromatic plants [83].

Different phenolic compounds were also identified using an eight-channel CoulArray detector in tea samples [84]. The applied potentials for the eight cells of the CoulArray detector ranged from +250 to +900 mV. 4-Hydroxycoumarin was the most abundant antioxidant compound in all 11 tea samples analyzed in the latter study. However, syringic and ferulic acids had the lowest concentrations among the phenolic compounds in the tea samples.

### 4.3. Beverages

Many bioactive compounds can be found in beverages due to the diverse components present in drinks. The phenolic compounds present in beverages originate from the plant or fruit used during their production, such as barley or hops for beer and honey for mead. A 16 channel CoulArray detector was used for the analysis of several phenolic compounds in juices and other beverages [85,86]. An HPLC-CAD with 8 porous graphite working electrodes was used for the analysis of phenolic compounds in wines and meads [87]. The potentials of the electrochemical cells were set in the range from +200 to +900 mV at +100 mV increments. The limit of detection for the phenolic compounds that were analyzed was 2.8–15.0 μg L^−1^. The compounds that were detected were derivatives of cinnamic and benzoic acid. Vanillin had the highest concentration among all the phenolic compounds from the mead samples (0.570–5.171 mg L^−1^). In white wines, the highest concentration among the phenolic compounds detected was for rutin (0.875–5.078 mg L^−1^). 

In another study performed by Kahoun et al., a CoulArray detector with eight channels was used to detect different phenolic compounds in mead samples, benzoic acid hydroxyderivatives being the most common phenolic compounds [88]. Vanillin and ethyl vanillin contributed significantly to the sensory attributes of the different mead samples. Vanillin is normally detected in trace amounts, as it originates from honey or propolis. Higher concentrations can indicate the addition of vanillin intentionally, as found in samples of almond meads in the previous study. The phenolic content in mead is based on its composition and storage conditions. Another study analyzed phenolic compounds and flavonoids in beer samples using 11 different columns with different stationary phases in an HPLC system coupled to an eight-channel CoulArray detector [84] The potentials of the electrodes were set from +250 to +900 mV.

A comparative study was undertaken by Bocchi et al. for the determination of several polyphenolic compounds in a brandy sample using UV and coulometric detectors coupled with HPLC [89]. The working electrodes were set from −150 to +900 mV. The sensitivity and selectivity of the phenolic compounds were greater using the CoulArray detector than the UV, as the CoulArray detector was able to monitor the responses of all the phenolic acids due to the low limit of detection (1–5 μg L^−1^). The content of the phenolic acids presented ranged from 3.1 to 140 μg L^−1^.

### 4.4. Cereals

Not much research has been undertaken regarding the determination of phenolic compounds in cereals using a coulometric detector. Polyphenols in cereals do not just act as antioxidant compounds but also play a role in the prevention of parasitic and pathogenic bacteria, which results in extended shelf-life and preservation. This may vary depending on the type of grain used, as phenolic compounds are mainly found in the bran or cortical layer of grains, such as in rice. Ferulic acid is a common phenolic compound that is mainly found in its bound form, as it is associated with the fibre content in the cereal [90]. However, the bioavailability of phenolic compounds is still being investigated, as some studies have shown that the phenolic compounds present in cereals may change when processed or cooked [91]. Most cereals are consumed after cooking in water or baking. For example, free phenolic compounds may be leached from pasta when boiled, as they are water-soluble. On the other hand, increased temperature through cooking may alter molecular structure and texture and can breakdown cellular components, resulting in an increase in bound phenolics in the food matrix [92]. More attention should be paid to the detection and characterization of phenolic compounds in raw and processed cereals using the CoulArray detector, as very few studies have been conducted.

Dvořáková et al. used HPLC coupled with an electrochemical eight-channel CoulArray detector to quantify free phenolic compounds in barley and malt extracts [93]. The potentials that were applied to the electrochemical cells were +250, +300, +400, +500, +600, +700, +800, and +900 mV. Seventeen phenolic compounds were identified and quantified from all the barley and malt varieties. They included (+)-catechin, (−)-epicatechin, esculin, umbeliferone, scopoletin, rutin, and quercetin, along with gallic, protocatechuic, p-hydroxyphenyl-acetic, vanillic, chlorogenic, caffeic, syringic, p-coumaric, ferulic, and sinapinic acid. Ferulic acid was the most abundant free phenolic compound that was present in barley and malt, with concentrations varying from 12.5 to 21.9 and 7.8 to 56.1 μg g^−1^ DW for barley and malt, respectively. Moreover, the concentration of catechin was related to the presence or absence of the hull in barley and malt. In hulled genotypes, the catechin content was 11.0–15.5 μg g^−1^ DW for barley and 0.9–5.9 μg g^−1^ DW for malt, which were lower in comparison to dehulled genotypes (15.0–17.0 μg g^−1^ DW for barley and 10.6–12.1 μg g^−1^ DW for malt).

An analysis of alkylresorcinols (AR) from different cereals using HPLC-CAD was undertaken by Ross and Kochhar [94]. Alkylresorcinols are phenolic lipids that can be found in the bran of barley, wheat, and rye kernels. The CoulArray detector had eight electrodes, which had their potentials set from 0 to +850 mV. The AR quantity differed between cereal samples, as the concentration in refined wheat flour samples ranged from 13–47 µg g^−1^, which differed from that of wholegrain flours (489–660 µg g^−1^).

### 4.5. Others

Spice plants—namely, parsley roots and leaves, celery roots and leaves, onion, and dill leaves—were analyzed to determine their antioxidant activities using HPLC coupled with a model 5600 CoulArray with an array of four detection cells [95]. The electrochemical detector had the working electrodes’ potentials set at +300, +500, +700, and +900 mV. The flow rate of the mobile phase was set at 0.75 mL min^−1^ at the gradient of the phenolic compounds for 51 min. Chlorogenic acid was identified as the largest elution peak and the celery leaves contained the most phenolic compounds among the various spice vegetables.

The determination of the phenolic compounds in tomatoes was undertaken by Schindler et al. using a 16-channel electrode CoulArray detector [96]. The potentials applied were between +50 to +750 mV with an LOD of 6 μg L^−1^, and the authors were able to detect several phenolic compounds (naringenin, rutin, ferulic acid, *p*-hydroxybenzaldehyde, and *p*-coumaric acid).

The analysis of flavonoids and polyphenols in almonds was undertaken using an HPLC-CAD with 13 working electrodes and potentials set from +60 to +720 mV with +60 mV increments [97]. A wide range of phenolic acids were identified and verified using LC-MS, including catechin, protocatechuic acid, epicatechin, and quercetin. The CoulArray detector has also been used to investigate the phenolic profile in oils [98,99]. Bayram et al. selected eight phenolics to be quantified from olive oil samples (caffeic acid, vanillic acid, ferulic acid, p-coumaric acid, oleuropein, tyrosol, hydroxytyrosol, and pinoresinol) using a 4 channel coulometric array detector with potentials set at +250, +400, +500, and +750 mV, respectively. In all the oil samples from the latter study, the concentration of the total phenolic compounds did not exceed 1.7 mg kg^−1^ oil, which was in accordance with a previously conducted study that found concentrations below this amount [100]. The use of an electrochemical detector for the analysis of phenolic compounds in oil is effective, reliable, and suitable for oil adulteration determination.

Based on the analytical information obtained from the literature and shown in Table 1, the detection limits vary based on the type of sample being analyzed. It is not surprising that complex samples contain different phenolic compounds and elute with different retention times. The majority of the authors who have used chromatographic methods have employed a linear gradient with a constant flow coupled with a CoulArray detector when analyzing phenolic compounds. The use of other detection methods, such as UV absorption, is still considered to be more common. Diode array detectors are also applied due to their ability to obtain complete UV–Vis spectra of the analytes and the possibility of selecting a specific wavelength. However, the main challenge when using these detectors is acquiring the best possible detection limits and quantitation, especially for complex chemical structures, such as phenolic compounds. The alternative approach is the application of CoulArray detectors or mass-spectrometry techniques. The use of mass spectrometry with HPLC allows the possibility of obtaining the phenolic fractions of different food matrixes with a broader linear range. Gas chromatography (GC) can also be applied for the determination and quantification of phenolic compounds and is more suitable if the phenolic compounds undergo derivatization to increase their volatility. However, the CoulArray detector is a very sensitive technique when it comes to the detection of phenolic compounds. Since phenolic compounds demonstrate electrochemical behavior, the CoulArray can use lower detection potentials and have lower detection limits than DAD and UV detection. On the other hand, mass spectrometry-related techniques are more selective, as more information about the molecular weight and structure can be obtained. The coupling of the CoulArray detector with mass spectrometry allows the CoulArray to work as a reactor. The combination of an electrochemical detector with mass spectrometry opens more doors to understanding redox potentials, the antioxidant activities of substances and individual phenolic compounds from complex food samples. 

**Table 1:** Examples of phenolic compounds detected in food and beverages by the coulometric array detector.

Food Product Type	Food Matrix	Method	Working Electrode and Potential Range	Recovery %	Reproducibility (Coefficient of Variation (CV))	Limit of Detection (LOD)	Compounds	Reference
Fruit	Currant, gooseberry	FIA-ECD	4 PGEs, +200 to +800 mV	N/A	N/A	N/A	N/A	[71]
Fruit	Bilberry, lingonberry, cloudberry, seabuckthorn berry	RP-HPLC, gradient elution	8 PGEs, 0 to +840 mV	76.4% to 153.8%	N/A	N/A	Gallic acid	[72]
							vanillic acid	
							caffeic acid	
							p-coumaric acid	
							Ferulic acid, myricetin, quercetin, isorhamnetin	
Fruit	Swiss chard	RP-HPLC, gradient elution	2 PGEs, −50 to +825 mV	N/A	0.06%–1.05% CV	1 ng mL^−1^	Gallic acid, vanillic acid, caffeic acid, p-coumaric acid, ferulic acid, myricetin, quercetin, p-OH-benzoic acid, proto-catechuic acid, chlorogenic acid, syringic acid, catechin, kaempferol	[74]
Fruit	Blue honeysuckle	RP-HPLC, gradient elution	12 PGEs, −80 to +800 mV	N/A	N/A	N/A	N/A	[101]
	Saskatoon berry							
	Chinese hawthorn							
Fruit	Honeysuckle berries	RP-HPLC, N/A	12 PGEs, N/A	N/A	N/A	N/A	Gallic acid, catalposide, rutin, resveratrol, quercitrin, chlorogenic acid	[75]
Fruit	Strawberries	RP-HPLC, gradient elution	8 PGEs, +100 to +800 mV	N/A	N/A	N/A	Catechin, cinnamic acid derivatives, anthocyanin derivatives	[78]
Herbs	*Moringa oleifera, Melissa officinalis, Fraxinus excelsior,* and other officinal plants	RP-HPLC, gradient elution	16 PGEs, −50 to +700 mV	N/A	1.5% to 2%	1.3 ± 0.1 µM	Chlorogenic acid, isoquercetin, phloretic acid, oleuropein, osivitexin, gallic acid, catechin, protocatechuic acid, and others	[42]
Beverages	Red and white wines, meads	RP-HPLC, gradient elution	8 PGEs, +200 to +900 mV	N/A	N/A	2.8 to 15.0 µg L^−1^	Cinnamic acid derivatives, benzoic acid derivatives, and others	[87]
Beverages	Meads	RP-HPLC, gradient elution	8 PGEs, +200 to +900 mV	N/A	N/A	4 to 29 µg L^−1^	Gallic acid, protocatechuic acid, gentisic acid, vanillic acid, caffeic acid, syringic acid, *p*-coumaric acid, and others	[88]
Beverages	Beer, tea	RP-HPLC, gradient elution	8 PGEs, +250 to +900 mV	N/A	N/A	1 to 5 µg L^−1^	4-Hydroxycoumarin, gallic acid, vanillic acid, rutin, caffeic acid, naringenin, and others	[84]
Cereals	Barley and malt extracts	RP-HPLC, gradient elution	8 PGEs, +250 to +900 mV	N/A	N/A	N/A	(+)-Catechin, (−)-epicatechin, esculin, umbeliferone, scopoletin, rutin, quercetin, and others	[93]
Cereals	Wholegrain wheat flour, wheat semolina. barley, rye bran, spelt, oats	RP-HPLC, gradient elution	8 PGEs, 0 to +850 mV	98.4% to 107.5%	0.8% to >10% CV,	1 ng g^−1^	Alkylresorcinols	[94]
					depending on the different homologues			
Spices	Parsley	RP-HPLC, gradient elution	4 PGEs, +300 to +900 mV	N/A	N/A	4.75 µg mL^−1^	Chlorogenic acid	[95]
	celery							
	onion							
	dill leaves							
Fruit	Tomatoes	RP-HPLC, gradient elution	16 PGEs, +50 to +750 mV	81.1% to 89.8 ±2.8%	N/A	3 to 13 µg mL^−1^	Naringenin, rutin, ferulic acid, *p*-hydroxybenzaldehyde, *p*-coumaric acid, and others	[96]
Fruit	Almonds	RP-HPLC, gradient elution	13 PGEs, +60 to +720 mV	N/A	1.24% to 5.17%	N/A	Catechin, procatechuic acid, epicatechin, quercetin, and others	[97]
Oils	Olive oil	RP-HPLC, gradient elution	4 PGEs, +250 to +750 mV	N/A	N/A	0.03 to 1.7 ng mL^−1^	Tyrosol, hydroxytyrosol, oleuropein, pinoresinol, caffeic acid, ferulic acid, vanillic acid, *p*-coumaric acid	[99]

## 5. Relation with Other Antioxidant Assays

As well as analysing phenolic compounds in food extracts, several studies also related the signal derived from the CoulArray with in vitro antioxidant assays. This started with research by Guo et al. in 1997 analysing phenolic compounds in fruit and vegetable extracts [102]. Twelve electrode detectors poised from 0 mV to +770 mV with +70 mV increments were applied to collect coulometric signals from fruit and vegetable samples. The results, including total and dominant peak height (μA/μL) and total and dominant peak area (μC μL^−1^), were correlated with the oxygen radical absorbance capacity (ORAC) assay. The results showed that, except for spinach as an outlier, the Pearson correlation coefficients were high between the ORAC assay and signals from the CoulArray (r ranging from 0.914 to 0.956).

This method was developed by Aaby et al. using eight electrodes with +100 mV increments from +100 mV to +800 mV. For comparison purposes, three antioxidant assays, including DPPH, FRAP, and ORAC, were performed with several antioxidant standards [103]. Data analysis was performed with both individual channel peak integration and cumulative peak areas from low to high. The covariance between signals from the CoulArray and the antioxidant assays was modelled with principal component analysis (PCA). The first three principal components could explain 74% of the total variance. From the score plot, the FRAP was close to the signal area detected at +300 mV (r = 0.79), while the ORAC and DPPH were highly related to the cumulative area from +100 mV to +400 mV (DPPH, r = 0.93) and to +800 mV (ORAC, r = 0.57). Compared to other post-column methods, the CoulArray detector provides an easier alternative for the screening of the antioxidant capacity of unknown compounds. In 2005, Aaby et al. applied the Coulometric method to analyse the antioxidants in the flesh and achenes of strawberries [78]. High correlations were found with a cumulative response at +800 mV with both FRAP and ORAC assays (r = 0.99 and 0.96, respectively). Such correlations between antioxidant assays and CoulArray signals were further studied with officinal herbs [42] and olive oil [99].

To reduce the time taken for the determination of the antioxidant assay with CoulArray, Kongwong et al. [30] developed a flow injection system with lettuce and correlated the results with the DPPH assay. With this method, after the injection of the sample, the lettuce extracts induced sharp peaks lasting less than 15 s. With a sample interval of around 30 s, the flow injection system could guarantee sample analysis in 1 min [27]. Potentials from +100 to +850 were used and the highest correlation with DPPH analysis was achieved with potentials between +400 to +750 mV (R^2^ > 0.97). With a flow injection CoulArray system, the antioxidant capacity can be measured swiftly compared to common spectrophotometer methods.

## 6. Conclusions

This article reviews applications of the CoulArray detector for the analysis of phenolic compounds in food. Due to the antioxidant activity of phenols, they are considered electroactive compounds that can be easily oxidized by contact with the electrode surface of the CoulArray. The CoulArray detector has therefore proven to be effective, sensitive, and competitive with spectrophotometric detectors for the analysis of phenolic and antioxidant compounds found in food. Moreover, it is compatible with the gradient elution method when coupled with HPLC. The interest in coulometric array detectors has increased in the past decade due to their precision and high selectivity. This means that sample preparation and clean-up procedures can be conducted easily, as the selectivity of the CoulArray allows limitations on the matrix and spectral interferences during analysis.

CoulArray detectors are being used more due to their capacity for coupling with HPLC systems and mass spectrometry detectors, allowing improved characterization of phenolic compounds. In addition to its low cost and fast response, the use of different applied potentials in the CoulArray detector allows improved identification and broad screening of phenolic compounds. More studies should still be undertaken considering the identification of phenolic compounds from different foods, as there is a lack of information in the literature on the application of the CoulArray detector for the identification and characterization of phenolic compounds in cereals, vegetables, and fruits.

## Data Availability

The data is available on request from the authors.
